# Influenza virus infection drives upregulation of CD84 across a broad range of immune cells

**DOI:** 10.1002/cti2.70087

**Published:** 2026-03-09

**Authors:** Xiaoxiao Jia, Isabelle JH Foo, Hayley A McQuilten, Jeremy Chase Crawford, Aira F Cabug, Deborah Gebregzabher, Janet Chou, Robert C Mettelman, Tanya Novak, Lee‐Ann Van de Velde, Ryan S Thwaites, Adrienne G Randolph, Paul G Thomas, Jianqing Xu, Zhongfang Wang, Katherine Kedzierska, Lukasz Kedzierski, Brendon Y Chua

**Affiliations:** ^1^ Department of Microbiology and Immunology Peter Doherty Institute for Infection and Immunity, University of Melbourne Melbourne Australia; ^2^ Department of Host‐Microbe Interactions St. Jude Children's Research Hospital Memphis TN USA; ^3^ Center for Infectious Disease Research St Jude Children's Research Hospital Memphis TN USA; ^4^ Division of Immunology Boston Children's Hospital, Harvard Medical School Boston MA USA; ^5^ Department of Anesthesiology, Critical Care, and Pain Medicine Boston Children's Hospital Boston MA USA; ^6^ Department of Anaesthesia Harvard Medical School Boston MA USA; ^7^ National Heart and Lung Institute Imperial College London London UK; ^8^ Center for Influenza Disease and Emergence Response (CIDER) Athens GA USA; ^9^ Shanghai Public Health Clinical Centre and Institutes of Biomedical Sciences, Key Laboratory of Medical Molecular Virology of Ministry of Education/Health, Shanghai Medical College Fudan University Shanghai China; ^10^ State Key Laboratory of Respiratory Disease & National Clinical Research Center for Respiratory Disease, Guangzhou Institute of Respiratory Health Guangzhou Medical University Guangzhou China; ^11^ Institute for Vaccine Research and Development (IVReD) Hokkaido University Sapporo Japan; ^12^ International Collaboration Unit, International Institute for Zoonosis Control Hokkaido University Sapporo Japan

**Keywords:** CD84, infection, influenza, OLAH, T cells

## Abstract

**Objectives:**

Our previous study in hospitalised patients infected with avian A(H7N9) influenza virus identified *CD84* amongst several genes associated with recovery. Yet, the correlation between CD84 and respiratory viral infection outcomes is far from established. We aimed to define CD84 dynamics in patient cohorts of respiratory disease and immune cell populations in influenza virus‐infected mice.

**Methods:**

Expression dynamics of *CD84* and association with previously identified correlates of severe and fatal respiratory disease outcomes, *OLAH* and *IL18R1*, were analysed in A(H7N9) and COVID‐19 patient cohorts across disease severities. Using mouse models of influenza virus infection, CD84 expression on immune cell subsets was analysed over the course of infection.

**Results:**

Elevated *CD84* levels in recovered A(H7N9) patients were accompanied by increased expression of genes for CD84‐associated adaptor proteins and other SLAM receptor family members. In these patients, high *CD84* expression persisted until discharge, while remaining low throughout the disease in patients that succumbed. We found inverse correlations between *CD84* with *OLAH* and *IL18R1* levels in our A(H7N9) cohort, and in hospitalised COVID‐19 patients across respiratory disease severities. In influenza virus‐infected mice, CD84 was upregulated on a broad range of immune cell populations, particularly on activated and influenza virus‐specific T‐cell populations and correlated with less disease severity.

**Conclusion:**

Our findings revealed the link between high CD84 expression in humans and recovery from respiratory viral infections. In mice, CD84 expression increased across a broad range of immune cell populations, with CD84 expression on activated T‐cell populations correlating with less severe disease.

## Graphical abstract

Our findings reveal a link between high CD84 expression in humans and recovery from respiratory viral infections. In influenza virus‐infected mice, CD84 is upregulated on a broad range of innate and adaptive immune cell populations, particularly on activated and influenza virus‐specific T‐cell populations and associated with less disease severity.

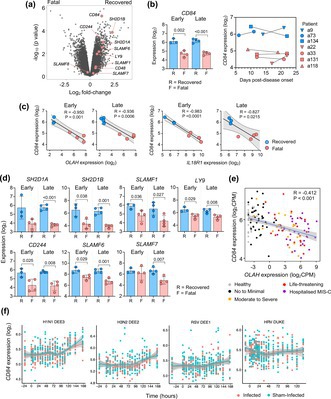

## Introduction

Respiratory viral infections are amongst the leading causes of death, with the young, elderly, pregnant women, individuals with co‐morbidities and indigenous populations being at the highest risk of developing severe adverse outcomes. Increased susceptibility to disease severity can also be underpinned by dysregulated immune responses, minimal pre‐existing immunity, as well as host genetic and metabolic factors. While the identification and use of reliable biomarkers predictive of severe outcomes can help to inform patient prognosis and treatment strategies, signatures of effective immune responses are similarly beneficial for predicting recovery from severe disease.

Our recent findings in hospitalised patients infected with avian‐derived A(H7N9), seasonal A/H1N1, SARS‐CoV‐2 and RSV identified key gene signatures associated with respiratory infection outcomes. High expression of genes encoding for oleoyl‐ACP hydrolase (*OLAH*) and IL‐18 receptor α chain (*IL18R1*) was strongly associated with life‐threatening respiratory complications and fatality.^1^, ^2^ In A(H7N9) infected patients, *CD84* was also amongst genes identified to be associated with recovery.^1^ However, to date, understanding how CD84 expression is linked to respiratory viral disease outcomes is far from clear.

CD84 is a member of the signalling lymphocytic activation molecule (SLAM) family of receptors, known for their role in the regulation of various immunological processes. CD84 is also known as SLAMF5, and like most other SLAM receptors, it functions as a self‐ligand and associates with intracellular adaptor proteins SAP (SLAM‐associated protein) and EAT‐2 (Ewing's sarcoma‐associated transcript 2) to mediate signalling. CD84 is expressed across immune cell populations, including T and B cells, myeloid cells, neutrophils and eosinophils, albeit at varying levels. Depending on the cell type and their activation state, diverse functional roles have been reported for CD84. Early studies indicated that ligation on T cells promotes proliferation^3^ and cytokine production.^4^ Expression on CD4^+^ T cells and B cells is important for stabilising cognate interactions,^5^ promoting plasma cell and germinal cell differentiation, and antibody production.^6^, ^7^ CD84 engagement can also mediate NK cell‐mediated cytotoxicity,^8^ autophagy and activation in antigen‐presenting cells,^9^, ^10^ and platelet aggregation.^11^ In addition, upregulation of CD84 has been found to be driven by Macrophage Migration inhibitory factor (MIF) secreted by multiple myeloma malignant cells.^12^ Previous studies have also found associations of CD84 SNP rs6427528 with improved responses to etanercept treatment in autoimmune diseases,^13^, ^14^ but mechanistic evidence for conferred protection remains elusive. CD84 has also been implicated in the development of cancers^15^, ^16^ and bacterial pathogenesis^17^; however, the association of CD84 expression with respiratory viral infections and disease outcomes remains unclear. Here, we aim to advance our understanding of how CD84 expression is tied to severe respiratory viral disease.

In our study, we provided evidence that in A(H7N9) patients who eventually recovered, CD84 expression persisted at high levels until hospital discharge. In contrast, CD84 levels remained low throughout the hospital stay in H7N9 patients with fatal disease outcomes. Moreover, our analysis revealed inverse correlations between *CD84* and *OLAH* as well as *IL18R1* levels in our hospitalised A(H7N9) and COVID‐19 cohorts across a range of respiratory disease severities. Using a mouse model of influenza virus infection, we found that CD84 was upregulated on a broad range of innate and adaptive immune cells over the course of disease. Increased CD84 expression was prominent on activated and influenza virus‐specific T‐cell populations, particularly on CD4^+^ T‐cell subsets, correlating with less disease severity. Overall, our findings demonstrated the link between CD84 expression in humans and recovery from respiratory viral infections, while our mouse influenza model showed increased CD84 expression levels on a broad range of immune cell populations, thus making CD84 a hallmark of immune activation and recovery.

## Results

### High *CD84* expression in patients hospitalised with influenza and COVID‐19 is associated with less severe respiratory disease outcomes

**Figure 1:**
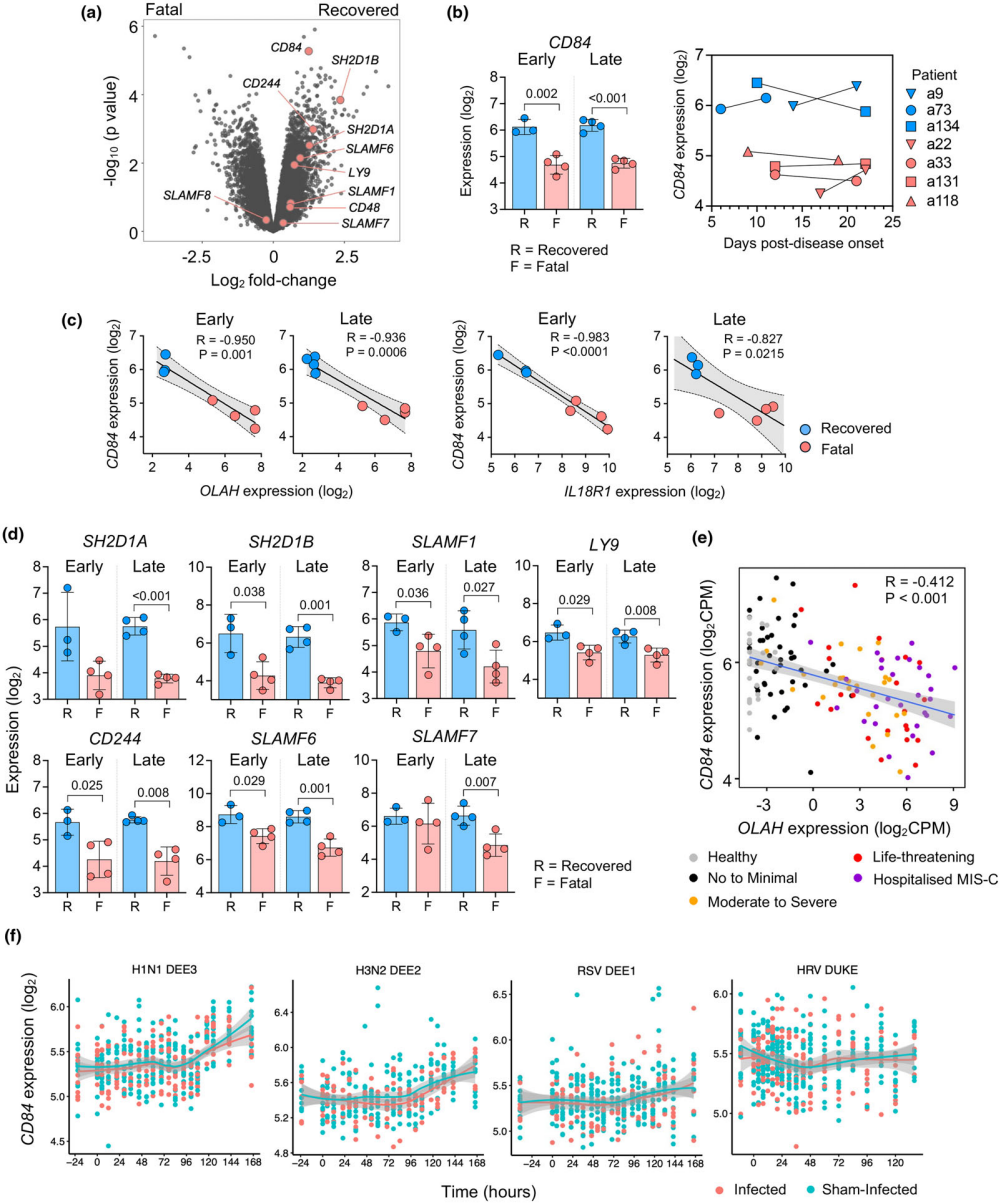
Elevated *CD84* expression is associated with recovery from severe A(H7N9) influenza and reduced COVID‐19 severity. Whole blood samples were collected from patients hospitalised with A(H7N9) at early (within 6 days of hospital admission) and late stages of disease (at discharge or 21–22 days post‐disease symptom onset). **(a)** Volcano plot of differentially expressed genes between fatal and recovered patients. Genes encoding for signalling lymphocytic activation molecule (SLAM) family receptors and adaptor proteins are indicated. **(b)** *CD84* transcript levels (mean ± SD) from early and late time points in fatal and recovered patients, with expression levels in each patient depicted over the course of disease. **(c)** Correlation between *CD84* and *IL18R1* or *OLAH* expression in fatal and recovered patients was assessed using Spearman's rank correlation (R). **(d)** Statistically significantly different transcript levels of genes encoding for SLAM family receptors and adaptor proteins (mean ± SD) between fatal and recovered patients at early and late time points. **(e)** Correlation between *CD84* and *OLAH* expression from RNA sequencing data of whole blood from healthy volunteers and hospitalised patients with acute COVID‐19 or MIS‐C; all aged < 21 years (*n* = 143). **(f)** *CD84* expression across time for human challenge models of mild respiratory infections (H1N1 DEE3 *n* = 477, H3N2 DEE2 *n* = 355, HRV Duke *n* = 471, RSV DEE1 *n* = 420) ranging from 24 h prior to and up to 170 h after infection. Smooth curves are fit using a LOESS model, with red corresponding to samples from infected subjects; blue corresponding to sham‐infected subjects. Linear mixed models were used to test for effects of time, infection status and the interaction of time with infection status, with subject included as a random effect. Infection status was not significant for any study.

In our previous studies, we investigated blood transcriptomic signatures associated with fatal influenza outcomes in hospitalised A(H7N9) patients.^1^, ^2^ Detailed information on these patient cohorts, including detailed demographics, is described in Jia et al.^2^ Our analyses revealed that *OLAH* and *IL18R1* levels were highly expressed early after hospitalisation in patients who had fatal disease outcomes compared to patients who recovered. Conversely, in the same A(H7N9) cohort, *CD84* was also amongst several genes identified to be associated with recovery.^1^ Here, we analysed the expression dynamics of genes encoding for CD84 as well as other SLAM receptor family members and their associated adaptor proteins throughout the course of disease in these patients (Figure 1a and 1b). Recovered A(H7N9) patients exhibited higher *CD84* levels following hospitalisation (early time points) which persisted until hospital discharge (late time points; Figure 1b). In contrast, in A(H7N9) patients who died, *CD84* expression remained low for the duration of their hospital stay. *CD84* levels also inversely correlated with *OLAH* and *IL18R1* levels in each patient at both time points (Figure 1c), known correlates of life‐threatening respiratory diseases. Genes encoding for SLAM‐associated adaptor protein *SH2D1B* (EAT‐2) as well as other SLAM family members; *SLAMF1, LY9* (SLAMF3), *CD244* (SLAMF4) and *SLAMF6* were also highly expressed in A(H7N9) recovered patients at both time points, while *SH2D1A* (SAP) and *SLAMF7* were significantly higher at the late time point (Figure 1d).

To further examine the link between *CD84* and *OLAH* expression, we analysed bulk RNA sequencing data from a cohort of young patients (aged 0–21) hospitalised with COVID‐19 and multisystem inflammatory syndrome in children (MIS‐C), in which *OLAH* expression was previously found to be markedly elevated in life‐threatening disease in comparison with mild symptoms.^2^ Our analysis revealed a strong inverse correlation between *CD84* and *OLAH* levels (Figure 1e), implicating elevated *CD84* expression is a hallmark of less severe respiratory viral disease. Importantly, we found no effects of age or sex on CD84 expression in this cohort (Supplementary figures 2a and 2b), and there was no significant correlation between CD84 expression and body mass index (BMI) of participants (Supplementary figure 2c).

We next investigated whether increased *CD84* expression was also associated with recovery from mild respiratory infections, by analysing transcriptomic data from human challenge models of mild H1N1, H3N2, human rhinovirus (HRV) and respiratory syncytial virus (RSV) infection.^18^ Baseline CD84 expression in participants prior to infection demonstrated no evidence of bimodal distribution, suggesting there are not individuals within the healthy population with particularly low or high CD84 expression (Supplementary figure 2d). Moreover, our analysis showed unaltered *CD84* expression across any of these mild respiratory virus infections compared to sham‐infection (Figure 1f), indicating elevated *CD84* expression is not a feature of relatively mild infections in limited viral exposure settings.

### CD84 expression increases across immune cell populations during influenza virus infection in mice

**Figure 2:**
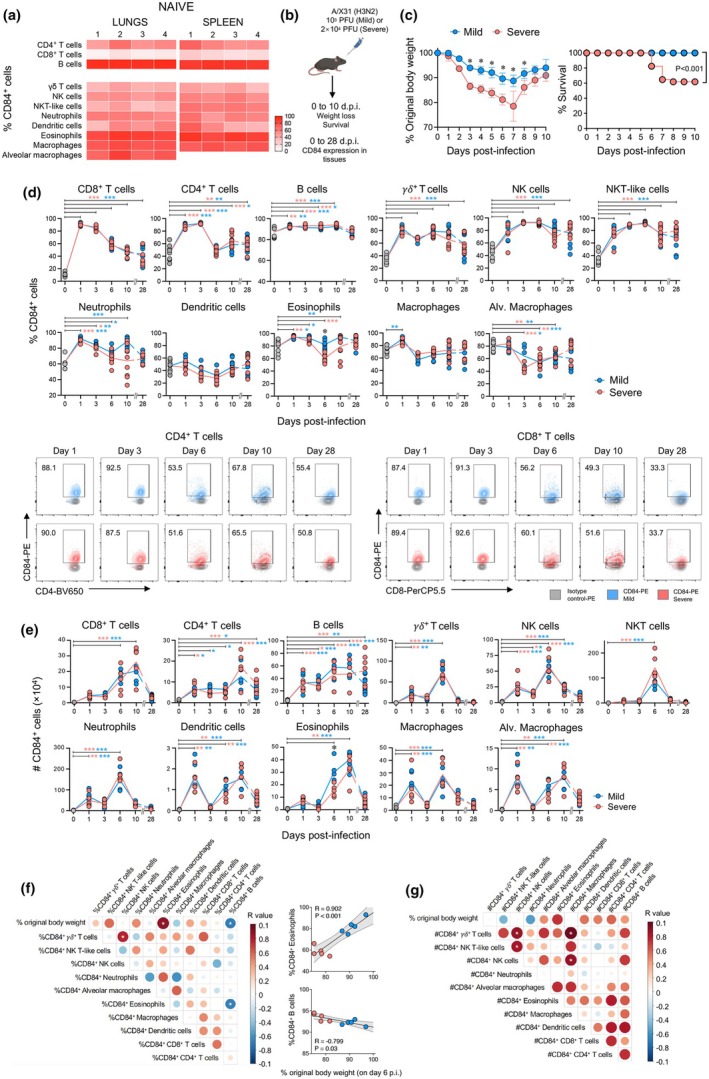
CD84 expression is upregulated across a broad range of immune cell populations in mice following influenza virus infection. **(a)** Heatmap showing the mean frequency of CD84^+^ cells on innate and adaptive immune cells in the lungs and spleen of naïve C57BL/6 mice (*n* = 4). **(b)** Mice were intranasally infected with a low (10^3^ pfu) or high (2 × 10^4^ pfu) dose of A/HKx31 to model mild and severe influenza disease, respectively (*n* = 5–10 per group at each time point). Analyses were performed at multiple time points following infection. **(c)** Body weight loss and survival were monitored daily for 10 days following infection. **(d)** Frequency and **(e)** numbers of CD84^+^ innate and adaptive immune cells in the lungs over the course of infection with representative dot plots of CD84 expression on lung CD4^+^ and CD8^+^ T‐cell populations. Statistical comparisons indicated are relative to baseline levels in **(a)** with blue or red asterisks referring to analysis of populations from mild or severe virus infections, respectively (*P < 0.05, **P < 0.01, ***P < 0.001). Black asterisks indicate statistical significance between infection doses. Correlation matrices between **(f)** frequencies and **(g)** numbers of CD84^+^ cells from infected mice with percentage original body weight on days post‐infection (6 d.p.i.). Positive or negative correlations are in shades of blue or red, respectively, with significance denoted by a white asterisk. Significant correlations with percentage original body weight for **(f)** are depicted in plots with each individual mouse shown and representative from two independent experiments. Statistical analysis was performed by **(c)** log‐rank (Mantel‐Cox) test for differences in survival and Mann–Whitney tests for weight loss differences on each day, **(d, e)** Mann–Whitney test and **(f, g)** Pearson correlation.

To further define the association between CD84 and respiratory viral infection outcomes, we analysed expression levels on innate and adaptive immune cells in a C57BL/6 mouse model of influenza A virus infection (Supplementary figure 1a, 1b and 1c). We first analysed CD84 expression in naïve mice and found that surface CD84 was expressed on a large proportion of eosinophils, macrophages and neutrophils (> 75%), and to a lesser extent on γδ T cells, dendritic cells, NKT‐like and NK cells (~30–55%) in both lungs and spleen (Figure 2a and Supplementary figure 1c). High CD84 expression was also found on the majority of B cells (> 90%) and a lesser proportion of CD4^+^ (~40%) and CD8^+^ T cells (~20%).

We established models of mild and severe influenza disease, as previously performed^1^, ^2^ by infecting mice with 10^3^ or 2 × 10^4^ pfu of A/HKx31 (H3N2), respectively (Figure 2b). Mice infected with the higher influenza virus dose lost more weight at 3–8 days post‐infection (d.p.i.), with 40% of mice succumbing to the disease by 7 d.p.i. (Figure 2c). Infection with either virus led to upregulation of CD84 to a similar extent on most cell populations in lungs (Figure 2d) and spleen (Supplementary figures 3a and 3b) within 10 d.p.i. Within the lung, higher levels of CD84 on eosinophils were observed at 6 d.p.i., with the milder dose and expression levels on dendritic cells and alveolar macrophages remaining unchanged or decreasing, respectively. While CD84 levels remained elevated in CD8^+^ and CD4^+^ T cells, γδ T cells, NK and NKT‐like cells, they returned to baseline levels across all immune populations by 28 d.p.i. This trend was largely reflected by similar increases in numbers of CD84^+^ immune populations over 10 days following infection with either infectious dose before subsiding by 28 d.p.i. (Figure 2e) and again typified by more CD84^+^ eosinophils associated with milder infection. Of note, biphasic peaks in numbers of CD84^+^ conventional and alveolar macrophages, neutrophils and dendritic cells were observed, with an initial peak on Day 1 followed by a second distinct rise on 6 d.p.i.

In examining relationships between CD84^+^ populations and disease severity at 6 d.p.i., we found less weight loss was associated with elevated frequencies of CD84^+^ eosinophils, and to a lesser extent, inversely correlated with CD84^+^ B cells (Figure 2f). Strong positive correlations were also found between frequencies and/or numbers of CD84^+^ γδ T cells, NK cells, NKT cells and macrophages (Figures 2f and 2g). Taken together, CD84 is unregulated across a wide range of immune cell populations in lungs during the acute phase of influenza virus infection.

### Elevated expression of CD84 is associated with activated and antigen‐specific T cells

**Figure 3:**
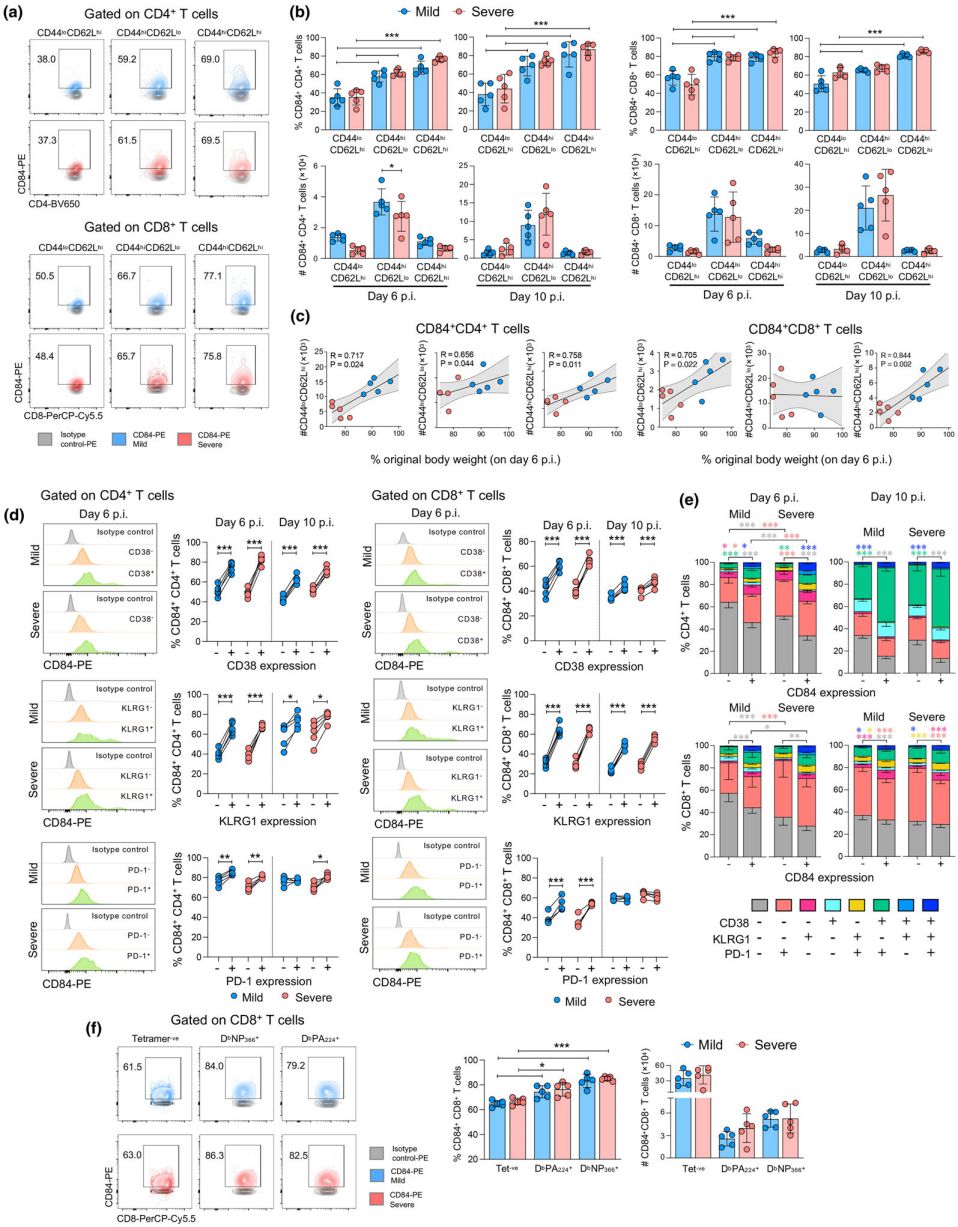
Increased CD84 expression on T cells is linked to their activation state. **(a)** Representative dot plots of CD84^+^ frequencies within lung CD4^+^ and CD8^+^ T‐cell populations with naïve (CD44^lo^CD62L^hi^), effector memory (CD44^hi^CD62L^lo^) and central memory‐like (CD44^hi^CD62L^hi^) phenotypes at 6 days post‐infection (d.p.i.). **(b)** Comparison of frequency (top) and numbers (bottom) of CD84^+^ naïve, effector and memory‐like T‐cell populations at 6 and 10 d.p.i. **(c)** Pearson's correlations between percentage original body weight and corresponding population numbers at 6 d.p.i. **(d)** Representative histograms showing expression of activation markers CD38, KLRG1 and PD‐1 on CD84^+^ and CD84^−^ CD4^+^ and CD8^+^ T cells in mild and severe disease groups (left) and comparison of their frequencies by paired *t*‐test, with lines denoting matched pairs (right). **(e)** Frequency of CD84^+^CD4^+^ and CD84^+^CD8^+^ T cells expressing none or one to three activation markers were determined using combinational Boolean gating. **(f)** Representative dot plots of CD84^+^ frequencies within lung influenza virus epitope‐specific D^b^PA_224_ and D^b^NP_366_ CD8^+^ T cells and tetramer negative (Tet^−^) populations (left), with bar graphs showing frequencies and numbers in mild and severe groups (right). (*P < 0.05, **P < 0.01, ***P < 0.001). Statistical analysis was performed by **(b, f)** Two‐way ANOVA with a Holms–Sidak post hoc test, **(c)** Pearson correlation, **(d)** One‐way or **(e)** two‐way repeated measures ANOVA with a Holms–Sidak *post hoc* test.

As T cells play an important role in influenza disease resolution, we analysed CD84 expression on T‐cell subsets in influenza virus‐infected mice based on their expression of CD44 and CD62L activation markers (Supplementary figure 4a). Our analysis revealed higher CD84 expression on effector (CD44^hi^CD62L^lo^) and central memory‐like (CD44^hi^CD62L^hi^) CD4^+^ and CD8^+^ T cells compared to populations with a naïve phenotype (CD44^lo^CD62L^hi^) populations in lungs (Figures 3a and 3b) and spleen (Supplementary figure 4b) at 6 and 10 d.p.i. Although there were no differences in frequencies of CD84^+^ T‐cell subsets between the viral doses, higher numbers of CD44^hi^CD62L^lo^ CD4^+^ T cells were detected during mild infection. Indeed, less weight loss was found to strongly correlate with increased numbers of all CD84 expressing CD4^+^ T‐cell subsets as well as CD44^hi^CD62L^hi^ and CD44^hi^CD62L^hi^ CD8^+^ T cells (Figure 3c).

Further analyses of activated CD4^+^ and CD8^+^ T cells based on increased expression of the activation markers CD38, PD‐1 and KLRG1 (Supplementary figure 1b) at these same time points showed that compared to CD38^−^, PD‐1^−^ and KLRG1^−^ populations, CD84 was significantly upregulated on populations expressing these markers (Figure 3d) and at a similar level between mice infected with either viral dose.

Frequencies of CD4^+^ and CD8^+^ T cells expressing none or one of these markers were also determined using combinational Boolean gating on CD84^−^ or CD84^+^ populations.^19^ In line with higher CD84 on activated T cells, populations of CD84^+^CD4^+^ and CD84^+^CD8^+^ T cells composed of lower proportions of CD38^−^PD‐1^−^KLRG1^−^ cells (grey) compared to CD84^−^ populations, and higher frequencies of phenotypes associated with activation (Figure 3e). Moreover, relative to the mild infection, there were lesser CD38^−^PD‐1^−^KLRG1^−^ cells in a severe infection, suggestive of a more activated and differentiated response.

Activated CD84^+^CD4^+^ T‐cell populations at 6 d.p.i. were mainly typified by higher prevalence of PD‐1 expressing phenotypes, with more PD‐1^+^ (orange) cells associated with severe infection. At 10 d.p.i., however, CD38^+^PD‐1^+^ (green) cells were the predominant population in both infection groups. In contrast, both CD84^−^ or CD84^+^ CD8^+^ T‐cell populations exhibited similar activation profiles at 6 d.p.i., although by 10 d.p.i., this response was again dominated by PD‐1 expressing phenotypes with no differences observed between infection doses.

As antigen‐specific CD8^+^ T cells are important for viral clearance following influenza virus infection, we also stained CD8^+^ T cells using immunodominant D^b^NP_366_ and D^b^PA_224_ tetramers to delineate differences in CD84 expression (Supplementary figure 1c). Compared to tetramer negative populations, both D^b^NP_366_^+^ and D^b^PA_224_^+^ CD8^+^ T cells in lungs (Figure 3f) and spleen (Supplementary figure 4d) elicited by either infection had similarly higher levels of CD84. Collectively, these data indicate that increased expression of CD84 within CD4^+^ and CD8^+^ T‐cell populations during influenza virus infection is associated with effector populations, activated phenotypes as well as antigen‐specific CD8^+^ T‐cell responses.

Finally, to investigate whether increased CD84 expression on T cells in infected mice was also reflected in humans, we utilised a patient cohort with scRNA‐seq data performed using PBMCs from healthy individuals and patients hospitalised with seasonal influenza and COVID‐19.^20^ Indeed, our analysis revealed CD84 expression in these patients across a broad range of cell types analysed, in particular CD4 and CD8 T cells (Supplementary figure 5), thus supporting our observations in our mouse models.

## Discussion

Hypercytokinemia, dysregulated and over‐activated immune responses, and organ damage are the hallmarks exhibited by influenza virus‐infected patients who experience life‐threatening complications that may eventually lead to fatal disease outcomes.^21^ Conversely, controlled and timely induction of inflammation and effective anti‐viral responses typify milder disease. Early prediction of these recovery versus death outcomes, ideally early in disease, would enable appropriate triage and implementation of tailored treatment strategies for patients at risk of dying.

Our previous studies in hospitalised A(H7N9) patients identified early and high expression levels of *OLAH* and *IL18R1* in patients with prolonged hospital stays who succumbed to fatal disease outcomes in comparison with patients who recovered.^1^, ^2^ In the present study, our findings identified a strong link between early *CD84* expression and subsequent recovery, revealing inverse correlations with *OLAH* and *IL18R1* levels in the same patients as well as in hospitalised COVID‐19 patients across a spectrum of respiratory disease severities. Early differences in expression levels of these genes therefore highlight their collective prognostic potential for differentiating patients who eventually recover or develop life‐threatening disease.

Although CD84 is known to be involved in many immunological processes and implicated in the development of autoimmunity, cancers and bacterial pathogenesis, CD84 expression levels in the context of respiratory viral infection outcomes are far from clear. Of note, a reported SNP in the CD84 gene (rs6427528) has been found to be associated with autoimmune conditions, including rheumatoid arthritis^13^ and psoriasis,^14^ which leads to higher CD84 gene expression and correlates with better treatment outcomes to anti‐TNF therapies, such as etanercept.

In line with our findings, upregulation of CD84 was also detected in blood of COVID‐19‐infected individuals experiencing mild symptoms and associated with shorter disease duration but absent in critical cases.^22^ *CD84* was also the most statistically significant gene upregulated amongst genes encoding for other members of the SLAM receptor family and together with elevated levels of SAP and EAT‐2 implicates SLAM‐mediated signal transduction in the regulation of immune responses during infection.

Our data from mice show upregulation of CD84 across a broad range of innate and adaptive immune cell populations during influenza virus infection, indicating CD84 may play a multifaceted role in the coordination of immune responses. CD84 is most recognised for its role in mediating adhesion between CD4^+^ T cells and B cells^5^, ^7^ as well as promoting T‐cell proliferation and cytokine production.^3^, ^4^ Indeed, our findings showed that high CD84 expression on both CD4^+^ and CD8^+^ T cells was associated with their activation and differentiation state as well as featuring prominently on influenza virus‐specific CD8^+^ T‐cell populations. Furthermore, correlations between CD84 expression levels on CD4^+^ T‐cell subsets and disease severity altogether highlighted a role for CD84‐mediated T‐cell responses in protection, supported by findings in influenza virus‐infected SAP‐deficient mice, which exhibited intrinsic CD4^+^ T‐cell defects in generating B‐cell responses and antibody production.^23^, ^24^ Of further relevance, our results are also in line with our previous study showing that recovery from infection within our H7N9 cohort was strongly associated with prominent CD4^+^ and CD8^+^ T‐cell responses.^25^ With elevated CD84 expression tied to this, and factoring in previous studies which demonstrated ligation of CD84 on T cells promotes proliferation and cytokine production,^3^, ^4^ our results here suggest a potential role for CD84 in promoting and regulating T‐cell‐mediated immunity to provide protection from severe respiratory virus infection in humans.

Prominent increases in CD84 expression were also observed on NK and NKT‐like cells, neutrophils and eosinophils over the course of infection. In particular, higher CD84 expression was observed on eosinophils during mild influenza virus infection, which may reflect their capacity to mediate more effective anti‐viral responses.^26^ Although several studies have described CD84‐mediated processes involving these populations, including promoting NK cell activation and cytotoxicity potential,^8^ NKT cell and eosinophilic development^27^, ^28^ and neutrophilic‐mediated regulation of immune responses,^29^, ^30^ it is important to note that the mechanisms and full extent of how this receptor modulates immune responses against influenza virus infection remain to be investigated in both mouse and human settings.

Our overall findings nonetheless provide insights into the potential role of CD84 in mediating protective immunity to influenza virus infection. We demonstrated that elevated CD84 expression constitutes a marker of immune activation, and our analysis of influenza and COVID‐19 patient cohorts strengthens the link between CD84 expression and recovery from respiratory viral infections. Our findings not only highlight its potential as a prognostic marker for disease progression and outcomes but also as a contributor to protection and/or rapid recovery from severe respiratory virus infection, providing a foundation for future studies to elucidate the direct role of CD84 in protection, its regulation in many different cell populations during infection and to develop strategies to increase CD84 expression.

## Methods

### Human and patient samples

Clinical details of patients infected with A(H7N9) influenza virus admitted to the Shanghai Public Health Clinical Center (SHAPHC) have been previously published.^2^, ^25^ Whole blood microarray (Affymetrix Human Gene ST‐2.0 arrays) was performed to identify differentially expressed genes (DEGs) from eight patients: four who recovered (Patients a73, a134, a20 and a9; mean age of 69) and were discharged within 14–23 days after disease onset, and four who succumbed (Patients a118, a33, a131 and a22; mean age of 70) at 19 days (Patient a118) or after prolonged hospital stays on Days 64, 70 and 76 (Patients a131, a33 and a22). Blood samples were collected at early (within 6 days of hospital admission) and late stages of disease (at 19–22 days post‐disease symptom onset).^2^ Informed consent was obtained from all participants, and the study was approved and conducted under supervision by the SHAPHC Ethics Committee.

For the COVID‐19 paediatric cohort, PAXgene whole blood samples were obtained from hospitalised patients aged < 21 years, recruited across US paediatric hospitals as part of the Overcoming COVID‐19 Study at Boston Children's Hospital (IRB‐P00033157). The demographics of this cohort are previously published.^2^ Blood samples were collected early after admission and RNA‐Seq data from these patients were analysed for *CD84* expression and correlation with expression of *OLAH*.^1^, ^2^ Patient disease severity was grouped according to degree of respiratory involvement comparing 43 participants with no to minimal respiratory dysfunction and requiring no major respiratory support other than oxygen or nebulisers, 25 with moderate‐to‐severe respiratory dysfunction requiring respiratory support with high flow nasal cannula oxygen or non‐invasive ventilation, and 23 with life‐threatening respiratory failure requiring invasive mechanical ventilation with some requiring extracorporeal membrane oxygenation (one died). As controls, samples from 22 uninfected, healthy individuals recruited at St Jude's Children's Research Hospital (Memphis, TN, USA) as part of the FLU09 cohort^31^ were also analysed.

*CD84* expression was also analysed across time in human challenge models of mild respiratory infections (H1N1 DEE3 *n* = 477, H3N2 DEE2 *n* = 355, HRV Duke *n* = 471, RSV DEE4 *n* = 420), using previously published datasets.^18^ *CD84* expression (probeset 211188_at) in each cohort was analysed using the same statistical methods models to control for factors and adjustments for multiple comparisons in the same patients and disease groupings as performed for the analysis of differential *OLAH* and *IL18R1* expression described previously.^1^, ^2^

### Influenza virus infection of mice

Mice were bred and maintained in the Biological Research Facility in the Department of Microbiology and Immunology at the University of Melbourne. All animal experimentation was conducted in accordance with the Australian National Health and Medical Research Council Code of Practice for the Care and Use of Animals for Scientific Purposes Guidelines and institutional regulations following approval (permit number: 20532) by the University of Melbourne Animal Ethics Committee. Influenza virus infection was performed under light anaesthesia with isofluorane and intranasal instillation (30 μL) with 10^3^ or 2 × 10^4^ plaque forming units (pfu) of A/HK/x31 (X31; H3N2). Mice were culled when a humane endpoint was reached; ≥ 25% of original body weight lost.

### Tissue sampling and preparation of single‐cell suspensions

All harvested tissues were passed through 70‐μm cell sieves to obtain single‐cell suspensions. Where necessary, cell suspensions were incubated with 0.15 M NH_4_Cl and 17 mM Tris‐HCI at pH 7.2 for 5 min at 37°C to lyse red blood cells. For lungs, enzymatic digestion in collagenase III (Worthington Biochemical Corporation; 2 mg/mL) and DNase I (Sigma‐Aldrich; 10 μg/mL) for 30 min at 37°C was performed prior to red blood cell lysis.

### Tetramer and antibody staining

Cells were stained with Fixable Live/Dead AquaBlue viability dye (Life Technologies) at room temperature for 10 min. Surface antibody staining was performed for 30 min at 4°C with various combinations of fluorochrome‐conjugated antibodies as specified in Supplementary figures 1a and 1b. For identification of influenza virus‐specific CD8^+^ T cells, cells were stained with D^b^NP_366–374_ (ASNENMETM) and D^b^PA_224–233_ tetramers (SSLENFRAYV) at room temperature in the dark for 1 h. Where required, cells were fixed in 1% paraformaldehyde for 20 min at 4°C prior to acquisition. Samples were acquired on a BD LSR Fortessa flow cytometer (Becton Dickinson), and data were analysed by the Flowjo Software version 10 (FlowJo LLC).

## Author contributions

**Xiaoxiao Jia:** Investigation; writing – review and editing. **Isabelle JH Foo:** Investigation; writing – review and editing. **Hayley A McQuilten:** Investigation; writing – review and editing. **Jeremy Chase Crawford:** Formal analysis; writing – review and editing. **Aira F Cabug:** Investigation; writing – review and editing. **Deborah Gebregzabher:** Investigation; writing – review and editing. **Janet Chou:** Resources; writing – review and editing. **Robert C Mettelman:** Resources; writing – review and editing. **Tanya Novak:** Resources; writing – review and editing. **Lee‐Ann Van de Velde:** Resources; writing – review and editing. **Ryan S Thwaites:** Resources; writing – review and editing. **Adrienne G Randolph:** Resources; writing – review and editing. **Paul G Thomas:** Resources; writing – review and editing. **Jianqing Xu:** Resources; writing – review and editing. **Zhongfang Wang:** Resources; writing – review and editing. **Katherine Kedzierska:** Supervision; conceptualization; investigation; project administration; writing – original draft; writing – review and editing. **Lukasz Kedzierski:** Supervision; conceptualization; investigation; project administration; writing – original draft; writing – review and editing. **Brendon Y Chua:** Supervision; conceptualization; investigation; project administration; writing – original draft; writing – review and editing.

## Conflict of interest

The authors declare no conflict of interest.

## Supporting information


Supplementary figure 1

Supplementary figure 2

Supplementary figure 3

Supplementary figure 4

Supplementary figure 5

## Data Availability

The data that support the findings of this study are available from the corresponding author upon reasonable request.

## References

[1] Cabug AF , Crawford JC , McQuilten HA *et al*. High expression of interleukin‐18 receptor alpha correlates with severe respiratory viral disease and defines T cells with reduced cytotoxic signatures. Nat Commun 2025; 16: 10344. 41285788 10.1038/s41467-025-65262-5 PMC12644894

[2] Jia X , Crawford JC , Gebregzabher D *et al*. High expression of oleoyl‐ACP hydrolase underpins life‐threatening respiratory viral diseases. Cell 2024; 187: 4586–4604. 39137778 10.1016/j.cell.2024.07.026

[3] Tangye SG , Nichols KE , Hare NJ , van de Weerdt BC . Functional requirements for interactions between CD84 and Src homology 2 domain‐containing proteins and their contribution to human T cell activation. J Immunol 2003; 171: 2485–2495. 12928397 10.4049/jimmunol.171.5.2485

[4] Martin M , Romero X , de la Fuente MA *et al*. CD84 functions as a homophilic adhesion molecule and enhances IFN‐gamma secretion: Adhesion is mediated by Ig‐like domain 1. J Immunol 2001; 167: 3668–3676. 11564780 10.4049/jimmunol.167.7.3668

[5] Cannons JL , Qi H , Lu KT *et al*. Optimal germinal center responses require a multistage T cell:B cell adhesion process involving integrins, SLAM‐associated protein, and CD84. Immunity 2010; 32: 253–265. 20153220 10.1016/j.immuni.2010.01.010 PMC2830297

[6] Burbage M , Gasparrini F , Aggarwal S *et al*. Tuning of *in vivo* cognate B‐T cell interactions by Intersectin 2 is required for effective anti‐viral B cell immunity. elife 2018; 7: 7. 10.7554/eLife.26556 PMC5770159 29337666

[7] Rao DA , Gurish MF , Marshall JL *et al*. Pathologically expanded peripheral T helper cell subset drives B cells in rheumatoid arthritis. Nature 2017; 542: 110–114. 28150777 10.1038/nature20810 PMC5349321

[8] Wang N , Calpe S , Westcott J *et al*. Cutting edge: The adapters EAT‐2A and ‐2B are positive regulators of CD244‐ and CD84‐dependent NK cell functions in the C57BL/6 mouse. J Immunol 2010; 185: 5683–5687. 20962259 10.4049/jimmunol.1001974 PMC3255554

[9] Agod Z , Pazmandi K , Bencze D *et al*. Signaling lymphocyte activation molecule family 5 enhances autophagy and fine‐tunes cytokine response in monocyte‐derived dendritic cells via stabilization of interferon regulatory factor 8. Front Immunol 2018; 9: 62. 29434592 10.3389/fimmu.2018.00062 PMC5790988

[10] Sintes J , Romero X , de Salort J , Terhorst C , Engel P . Mouse CD84 is a pan‐leukocyte cell‐surface molecule that modulates LPS‐induced cytokine secretion by macrophages. J Leukoc Biol 2010; 88: 687–697. 20628063 10.1189/jlb.1109756 PMC6608011

[11] Nanda N , Andre P , Bao M *et al*. Platelet aggregation induces platelet aggregate stability via SLAM family receptor signaling. Blood 2005; 106: 3028–3034. 16037392 10.1182/blood-2005-01-0333

[12] Lewinsky H , Gunes EG , David K *et al*. CD84 is a regulator of the immunosuppressive microenvironment in multiple myeloma. JCI Insight 2021; 6: 141683. 33465053 10.1172/jci.insight.141683 PMC7934939

[13] Cui J , Stahl EA , Saevarsdottir S *et al*. Genome‐wide association study and gene expression analysis identifies CD84 as a predictor of response to etanercept therapy in rheumatoid arthritis. PLoS Genet 2013; 9: e1003394. 23555300 10.1371/journal.pgen.1003394 PMC3610685

[14] van den Reek J , Coenen MJH , van de L'Isle Arias M *et al*. Polymorphisms in CD84, IL12B and TNFAIP3 are associated with response to biologics in patients with psoriasis. Br J Dermatol 2017; 176: 1288–1296. 27564082 10.1111/bjd.15005

[15] Rabani S , Gunes EG , Gunes M *et al*. CD84 as a therapeutic target for breaking immune tolerance in triple‐negative breast cancer. Cell Rep 2024; 43: 114920. 39466774 10.1016/j.celrep.2024.114920

[16] Zhu Y , Murtadha M , Liu M *et al*. Identification of CD84 as a potent survival factor in acute myeloid leukemia. J Clin Invest 2025; 135: e176818. 40198133 10.1172/JCI176818 PMC12126229

[17] Zheng N , Fleming J , Hu P *et al*. CD84 is a suppressor of T and B cell activation during mycobacterium tuberculosis pathogenesis. Microbiol Spectrum 2022; 10: e0155721. 10.1128/spectrum.01557-21 PMC8865571 35196822

[18] Liu TY , Burke T , Park LP *et al*. An individualized predictor of health and disease using paired reference and target samples. BMC Bioinformatics 2016; 17: 47. 26801061 10.1186/s12859-016-0889-9 PMC4722633

[19] Foo IJ , Chua BY , Clemens EB *et al*. Prior infection with unrelated neurotropic virus exacerbates influenza disease and impairs lung T cell responses. Nat Commun 2024; 15: 2619. 38521764 10.1038/s41467-024-46822-7 PMC10960853

[20] Mudd PA , Crawford JC , Turner JS *et al*. Distinct inflammatory profiles distinguish COVID‐19 from influenza with limited contributions from cytokine storm. Sci Adv 2020; 6: eabe3024. 33187979 10.1126/sciadv.abe3024 PMC7725462

[21] Nguyen THO , Rowntree LC , Chua BY , Thwaites RS , Kedzierska K . Defining the balance between optimal immunity and immunopathology in influenza virus infection. Nat Rev Immunol 2024; 24: 720–735. 38698083 10.1038/s41577-024-01029-1

[22] Patel H , Ashton NJ , Dobson RJB *et al*. Proteomic blood profiling in mild, severe and critical COVID‐19 patients. Sci Rep 2021; 11: 6357. 33737684 10.1038/s41598-021-85877-0 PMC7973581

[23] Elsner RA , Ernst DN , Baumgarth N . Single and coexpression of CXCR4 and CXCR5 identifies CD4 T helper cells in distinct lymph node niches during influenza virus infection. J Virol 2012; 86: 7146–7157. 22532671 10.1128/JVI.06904-11 PMC3416343

[24] Kamperschroer C , Dibble JP , Meents DL , Schwartzberg PL , Swain SL . SAP is required for Th cell function and for immunity to influenza. J Immunol 2006; 177: 5317–5327. 17015717 10.4049/jimmunol.177.8.5317

[25] Wang Z , Wan Y , Qiu C *et al*. Recovery from severe H7N9 disease is associated with diverse response mechanisms dominated by CD8(+) T cells. Nat Commun 2015; 6: 6833. 25967273 10.1038/ncomms7833 PMC4479016

[26] Samarasinghe AE , Melo RC , Duan S *et al*. Eosinophils promote antiviral immunity in mice infected with influenza a virus. J Immunol 2017; 198: 3214–3226. 28283567 10.4049/jimmunol.1600787 PMC5384374

[27] Huang B , Gomez‐Rodriguez J , Preite S , Garrett LJ , Harper UL , Schwartzberg PL . CRISPR‐mediated triple knockout of SLAMF1, SLAMF5 and SLAMF6 supports positive signaling roles in NKT cell development. PLoS One 2016; 11: e0156072. 27258160 10.1371/journal.pone.0156072 PMC4892526

[28] Jorssen J , Van Hulst G , Mollers K *et al*. Single‐cell proteomics and transcriptomics capture eosinophil development and identify the role of IL‐5 in their lineage transit amplification. Immunity 2024; 57: 1549–1566. 38776917 10.1016/j.immuni.2024.04.027

[29] Gong HH , Worley MJ , Carver KA , Godin CJ , Deng JC . Deficient neutrophil responses early in influenza infection promote viral replication and pulmonary inflammation. PLoS Pathog 2025; 21: e1012449. 39823516 10.1371/journal.ppat.1012449 PMC11845034

[30] Pettinella F , Mariotti B , Lattanzi C *et al*. Surface CD52, CD84, and PTGER2 mark mature PMN‐MDSCs from cancer patients and G‐CSF‐treated donors. Cell Rep Med 2024; 5: 101380. 38242120 10.1016/j.xcrm.2023.101380 PMC10897522

[31] Oshansky CM , Gartland AJ , Wong SS *et al*. Mucosal immune responses predict clinical outcomes during influenza infection independently of age and viral load. Am J Respir Crit Care Med 2014; 189: 449–462. 24308446 10.1164/rccm.201309-1616OC PMC3977720

