# Supplementary material for: Influenza virus infection drives upregulation of CD84 across a broad range of immune cells
Source: Clin Transl Immunology. 2026 Mar 9;15(3):e70087. doi: 10.1002/cti2.70087 (PMC12971607; doi:10.1002/cti2.70087)
Supplement: Supplementary file 1 — Supplementary figure 1 [file CTI2-15-e70087-s004.pdf]

Supplementary Figure 1

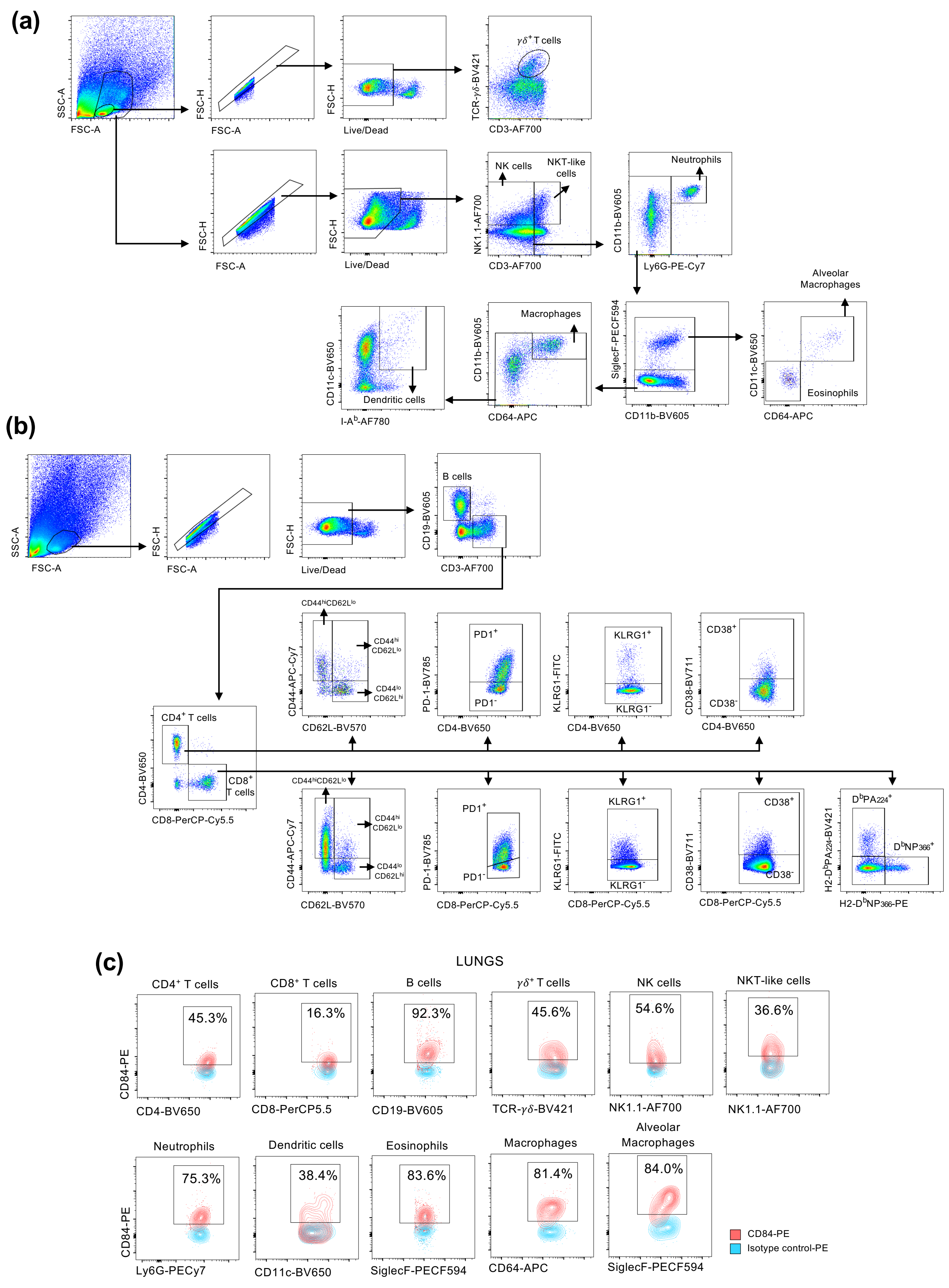

Supplementary Figure 1. Gating strategy for surface CD84 expression in mouse immune cell subsets. Representative gating strategy for identification of **(a)** innate and **(b)** adaptive immune cells. **(c)** Representative dot plots showing steady state expression levels of CD84 on naïve mouse immune cell subsets in the lungs.
