# Supplementary material for: Influenza virus infection drives upregulation of CD84 across a broad range of immune cells
Source: Clin Transl Immunology. 2026 Mar 9;15(3):e70087. doi: 10.1002/cti2.70087 (PMC12971607; doi:10.1002/cti2.70087)
Supplement: Supplementary file 2 — Supplementary figure 2 [file CTI2-15-e70087-s005.pdf]

Supplementary Figure 2

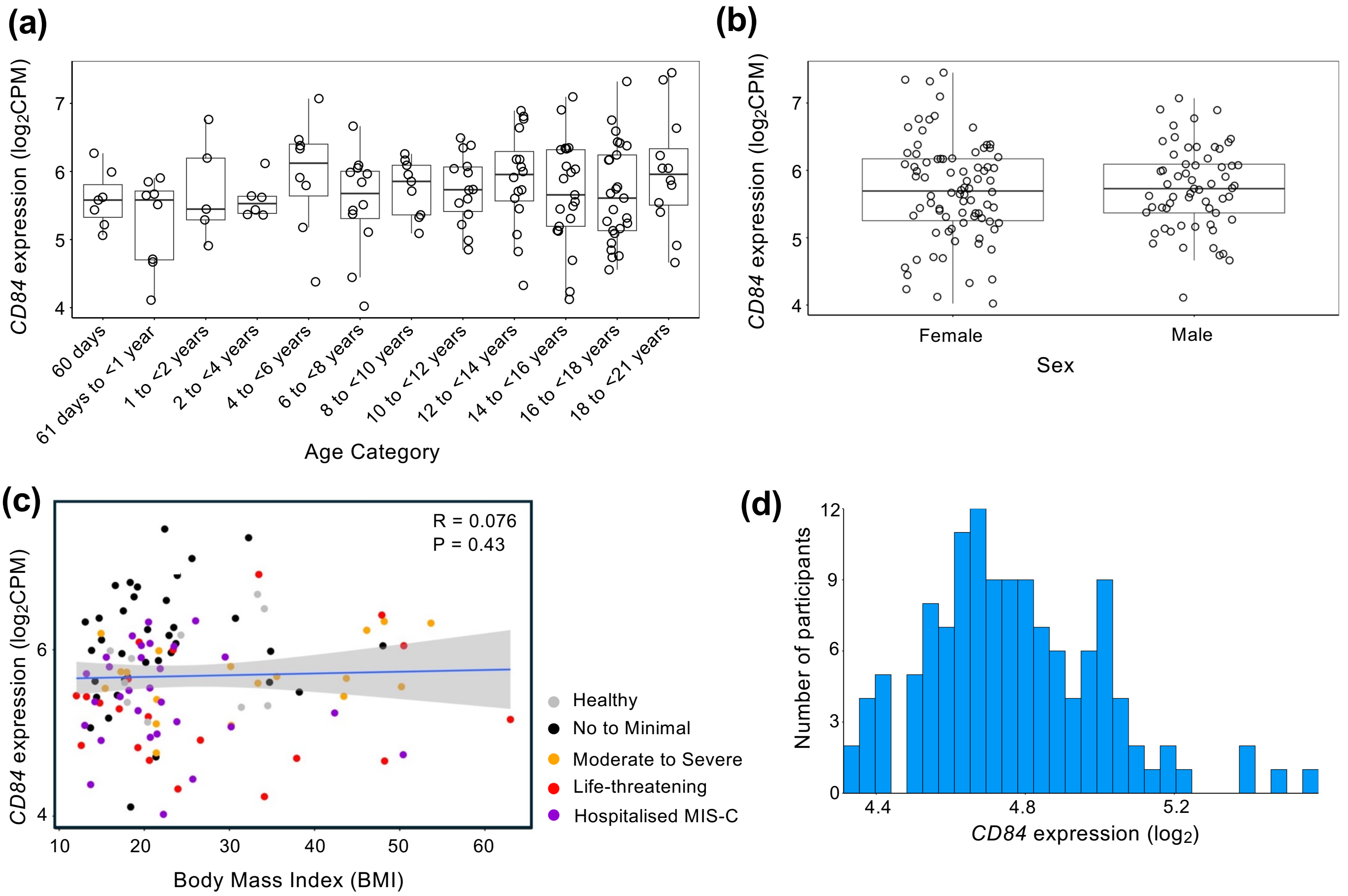

Supplementary Figure 2. CD84 expression in SARS-CoV-2 patients stratified by age, sex, correlation with body mass index (BMI) and distribution of CD84 expression in healthy participants. Analysis of CD84 expression from Figure **1e** across **(a)** age categories (*n* = 143) and **(b)** stratified by sex (female = 82 samples, male = 61 samples). **(c)** Correlation of CD84 expression with commonly used BMI ranges (<18.5 = underweight; 18.5–24.9 = healthy weight; 25–29.9 = overweight; 30–30.9 = obese; R40 = severely obese). This analysis only includes 117 participants for whom BMI data were available. **(d)** Analysis of CD84 expression in healthy participants enrolled in human challenge cohorts in Figure **1f** and additional 3 cohorts in Liu et al (20) (n=272). Hartigan's Dip Test indicate distribution of CD84 expression in these healthy individuals is not bimodal (p = 0.8194).
