# Supplementary material for: Influenza virus infection drives upregulation of CD84 across a broad range of immune cells
Source: Clin Transl Immunology. 2026 Mar 9;15(3):e70087. doi: 10.1002/cti2.70087 (PMC12971607; doi:10.1002/cti2.70087)
Supplement: Supplementary file 3 — Supplementary figure 3 [file CTI2-15-e70087-s003.pdf]

Supplementary Figure 3

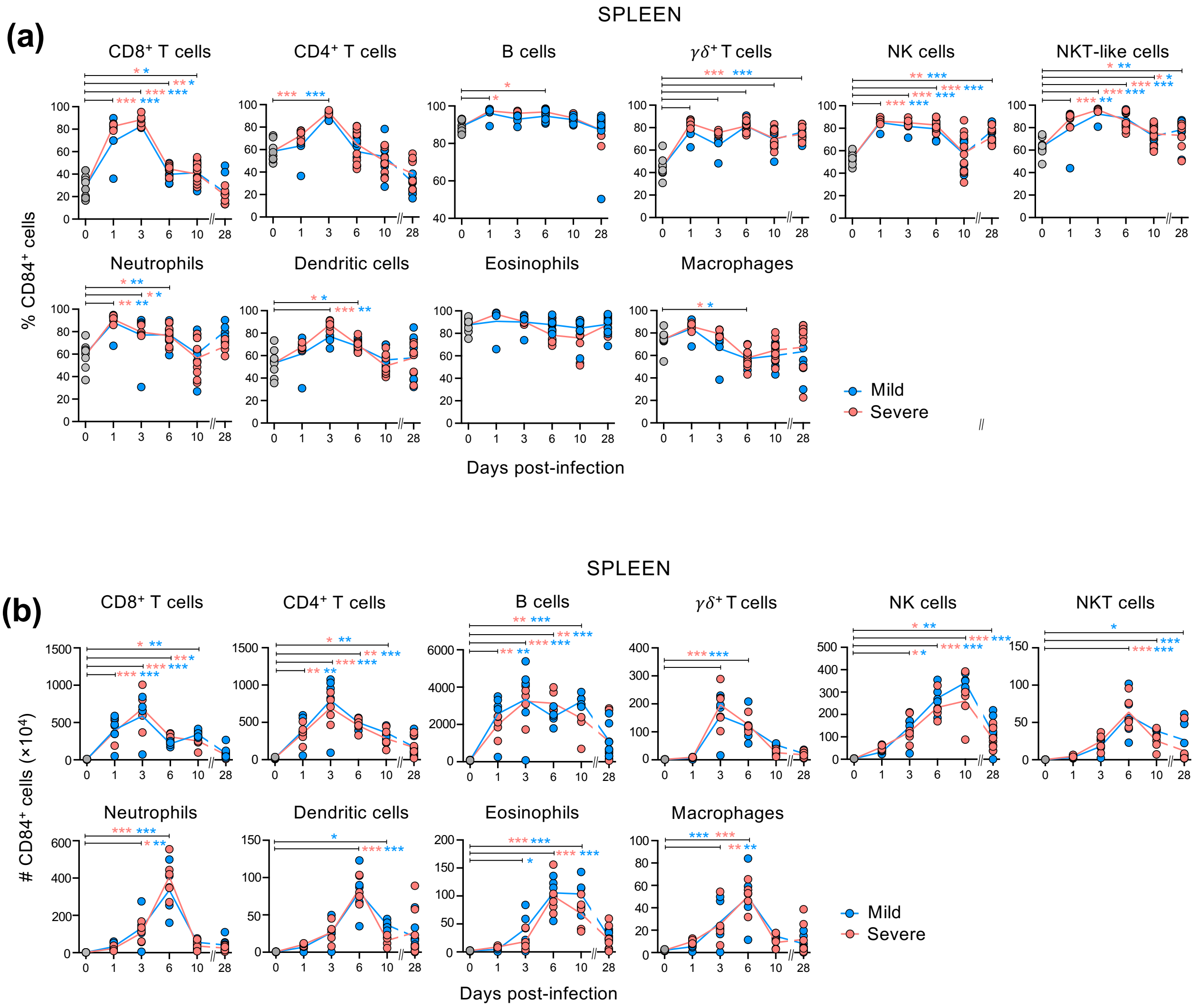

Supplementary Figure 3. Upregulation of CD84 expression on mouse spleen immune cell subsets following influenza virus infection.

(a) Frequency and **(b)** numbers of CD84⁺ innate and adaptive immune cells in the spleen over the course of infection with a low (10^3 pfu) or high (2×10^4 pfu) dose of A/HKx31 (n=5-10 per group at each time point). Statistical comparisons indicated are relative to baseline levels in Figure **1a**, with blue or red asterisks referring to analysis of populations from mild or severe virus infections, respectively (*P<0.05, **P<0.01, ***P<0.001). Black asterisks indicate statistical significance between infection doses. **(a, b)** Statistical analysis was performed by Mann-Whitney Tests.
