# Supplementary material for: Influenza virus infection drives upregulation of CD84 across a broad range of immune cells
Source: Clin Transl Immunology. 2026 Mar 9;15(3):e70087. doi: 10.1002/cti2.70087 (PMC12971607; doi:10.1002/cti2.70087)
Supplement: Supplementary file 4 — Supplementary figure 4 [file CTI2-15-e70087-s002.pdf]

Supplementary Figure 4

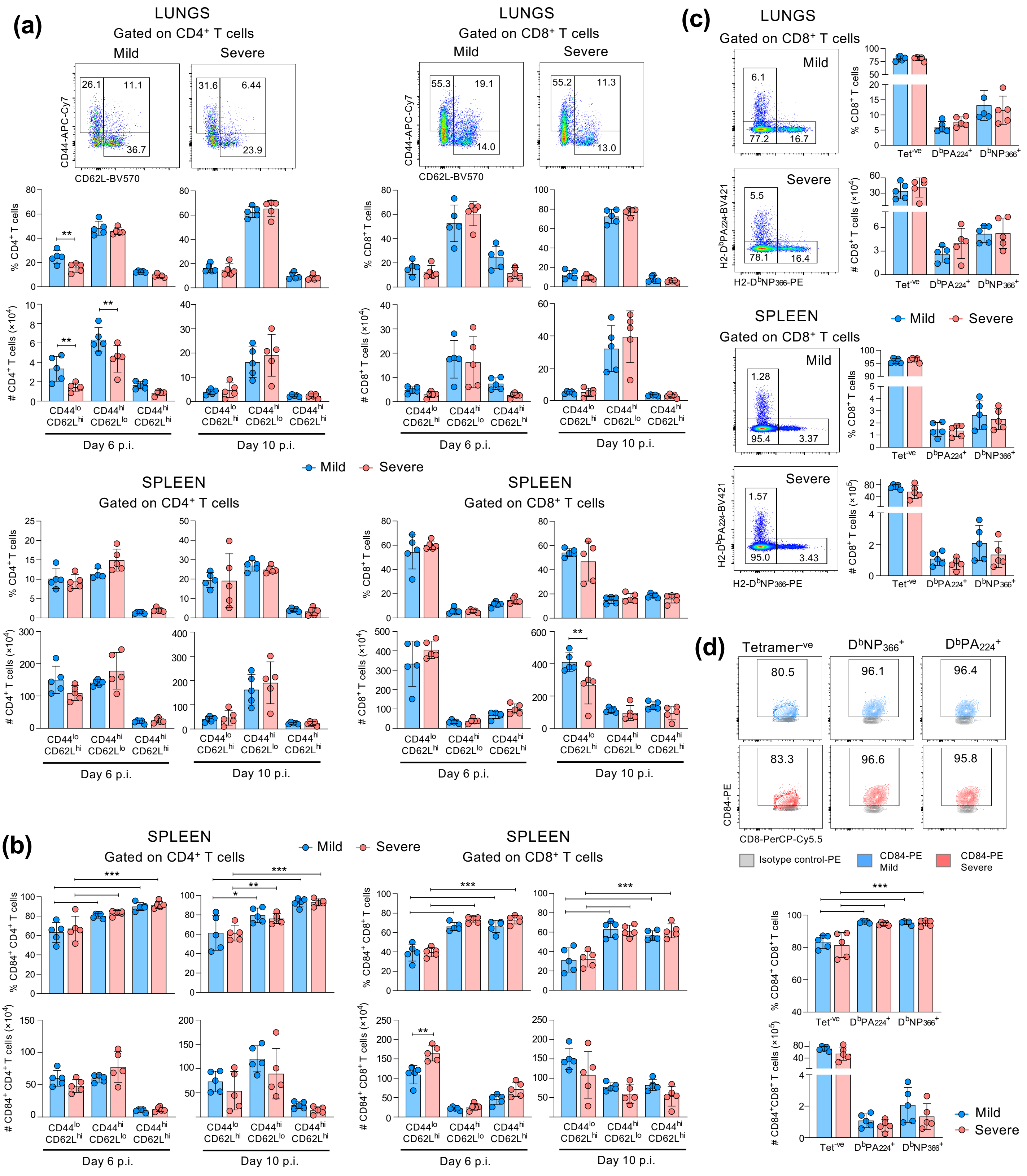

Supplementary Figure 4. Elevated CD84 expression on activated and antigen-specific T cell populations in the spleen. (a) Representative dot plots of CD44 and CD62L expression on lung $CD4^+$ and $CD8^+$ T cell populations at 6 d.p.i. (top). Bar graphs (below) show frequency and numbers naïve ($CD44^{lo}CD62L^{hi}$), effector ($CD44^{hi}CD62L^{lo}$) and memory-like ($CD44^{hi}CD62L^{hi}$) T cell populations in lungs and spleen at 6 and 10 d.p.i.. **(b)** Comparison of frequency (top) and numbers (bottom) of $CD84^+$ naïve, effector and memory-like T cell populations in the spleen. **(c)** Representative dot plots of D^bPA_{224} and D^bNP_{366} $CD8^+$ T cells in lungs and spleen at 10 d.p.i. with bar graphs depicting their frequencies and numbers. **(d)** Representative dot plots of $CD84^+$ frequencies within splenic influenza virus epitope-specific D^bPA_{224} and D^bNP_{366} $CD8^+$ T cells and tetramer negative (Tet^-) populations (above), with bar graphs showing frequencies and numbers (below). (*$P<0.05$, **$P<0.01$, ***$P<0.001$). **(a-d)** Statistical analysis was performed by Two-way ANOVA with a Holms-Sidak post-hoc test.
