# Supplementary material for: Influenza virus infection drives upregulation of CD84 across a broad range of immune cells
Source: Clin Transl Immunology. 2026 Mar 9;15(3):e70087. doi: 10.1002/cti2.70087 (PMC12971607; doi:10.1002/cti2.70087)
Supplement: Supplementary file 5 — Supplementary figure 5 [file CTI2-15-e70087-s001.pdf]

Supplementary Figure 5

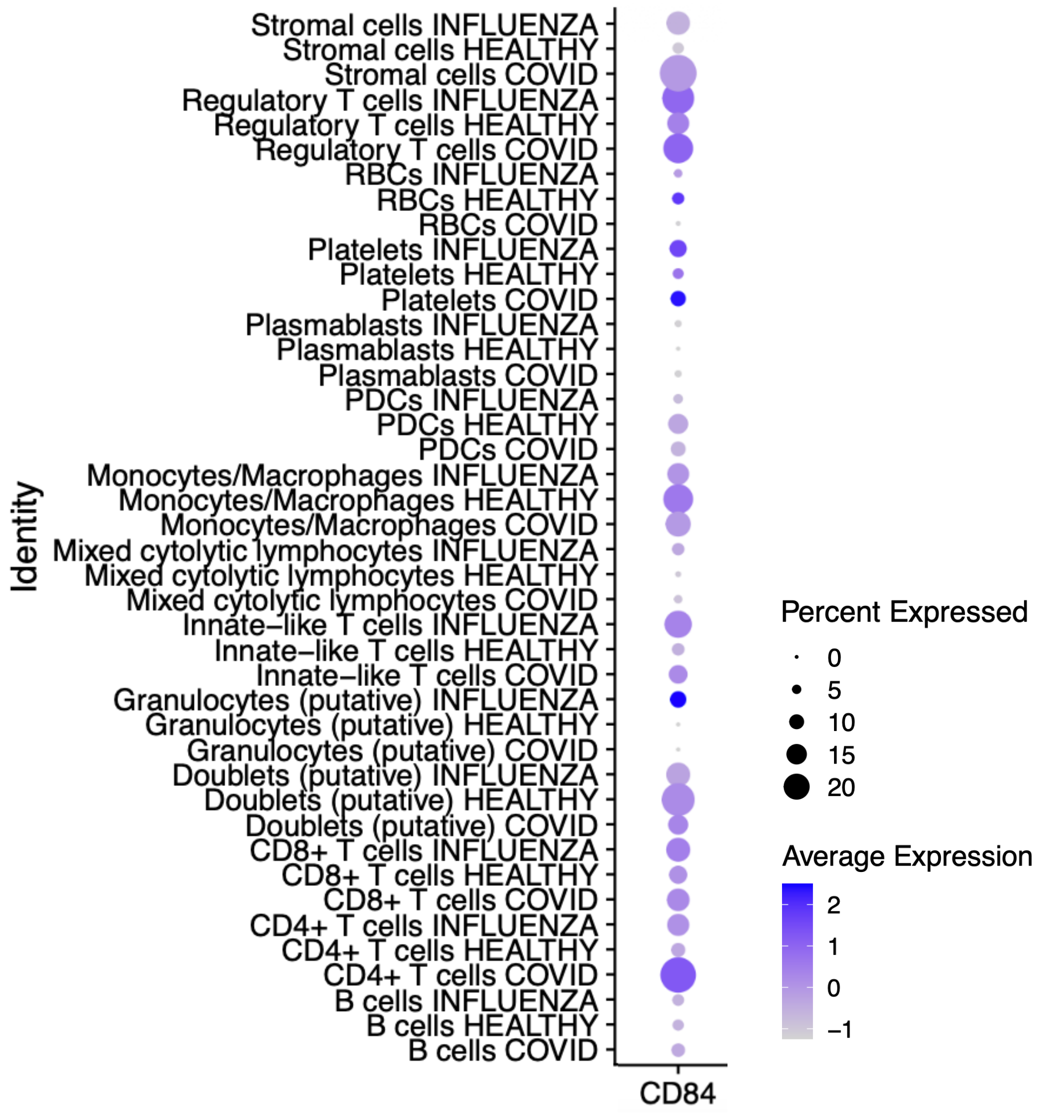

Supplementary Figure 5. Elevated CD84 expression in T cells from hospitalised influenza and COVID-19 patients. CD84 expression in single-cell RNA transcriptomic data acquitted from PBMCs of healthy individuals (n=2) or patients hospitalised with influenza (n=3) or COVID-19 (n=3) at 14 days post-symptom onset (22). Expression levels are segregated by disease state and cell population.
